# Outcomes of 3-D corrective osteotomies for paediatric malunited both-bone forearm fractures

**DOI:** 10.1177/17531934211029511

**Published:** 2021-07-14

**Authors:** Kasper C. Roth, Eline M. van Es, Gerald A. Kraan, Jan A. N. Verhaar, Filip Stockmans, Joost W. Colaris

**Affiliations:** 1 Department of Orthopaedics and Sports Medicine, Erasmus University Medical Centre, Rotterdam, The Netherlands; 2 Department of Orthopaedics, Reinier HAGA Orthopaedic Centre, Zoetermeer, The Netherlands; 3 Faculty of Medicine, KU Leuven KULAK, Leuven, Belgium

**Keywords:** Corrective osteotomy, malunion, fracture, forearm, radius, paediatric

## Abstract

Closed treatment of paediatric diaphyseal forearm fractures carries the risk of re-displacement, which can lead to symptomatic malunions. This is because growth will not correct angulation deformity as it does in metaphyseal fractures. The purpose of this prospective cohort study was to evaluate the outcomes after 3-D-planned corrective osteotomy with patient-specific surgical guides for paediatric malunited forearm fractures causing impaired pro-supination. Our primary outcome measure was the gain in pro-supination at 12 months follow-up. Fifteen patients with a mean age at trauma of 9.6 years and time until osteotomy of 5.9 years were included. Preoperatively, patients displayed a mean pro-supination of 67° corresponding to 44% of the contralateral forearm. At final follow-up, this improved to 128°, achieving 85% of the contralateral side. Multivariate linear regression analysis revealed that predictors of greater functional gain after 3-D corrective osteotomy are severe preoperative impairment in pro-supination, shorter interval until 3-D corrective osteotomy and greater angulation of the radius.

**Level of evidence:** III

## Introduction

Diaphyseal forearm fractures account for 15% of paediatric fractures (Cheng et al., 1999). Closed reduction and cast immobilization continue to be a major treatment method due to the great remodelling ability of paediatric fractures (Yang et al., 2012). However, fracture re-displacement occurs in 34% of displaced diaphyseal both-bone forearm fractures in children (Colaris et al., 2013), leading to malunion and decreased forearm rotation (Colaris et al., 2014), which may need a corrective osteotomy (Roth et al., 2017). Previously, a corrective osteotomy was indicated when pro-supination was less than 50–60% of the contralateral side (van Geenen and Besselaar, 2007). Price and Knapp (2006) recommended performing corrective osteotomy in forearm shaft malunions as soon as possible for angulations greater than 30° and to wait at least 6 months in malunions with angulations of 20–30° (Price and Knapp, 2006).

A corrective osteotomy is challenging due to angular deformities of both radius and ulna in coronal, sagittal. and axial planes (Bauer et al., 2017; Miyake et al., 2012). 3-D planned corrective osteotomy can aid in accurate correction of forearm malunions (Walenkamp et al., 2015). Using this method, patient-specific drilling and cutting guides can be 3-D printed to transfer the planned osteotomy plane to the patient’s bones during surgery.

The purpose of this prospective cohort study was to evaluate the clinical outcomes after 3-D-planned corrective osteotomy using patient-specific guides for malunited diaphyseal both-bone forearm fractures sustained during childhood. Our main research questions were what gain in forearm rotation can be achieved after 3-D corrective osteotomy for paediatric malunited forearm fractures and which factors are associated with greater functional gain.

## Methods

This cohort study was performed at a tertiary referral hospital (Erasmus University Medical Center, Rotterdam, The Netherlands). Ethical approval for this study was obtained from the Medical Ethical Testing Committee (reference number 52987.078.15). Written informed consent was obtained from all participants and their parents before the study. Our research protocol was registered in the National Trial Register (reference number 6324). This study was reported according to the guidelines by the strengthening the reporting of observational studies in epidemiology (STROBE) statement (von Elm et al., 2008).

### Participants

Inclusion criteria were a forearm malunion after a diaphyseal both-bone forearm fracture sustained during childhood (<18 years) and resulting in pronation or supination of <50° with unsatisfactory improvement after conservative treatment and a minimum age of 10 years at time of injury. Diaphysis was defined as the segment of the bone between 20% and 80% of its entire length (Nagy et al., 2008). Exclusion criteria were a traumatic osseous deformity of the contralateral forearm and a congenital or developmental deformity of the contralateral or affected forearm (such as radial or ulnar longitudinal deficiency, radioulnar synostosis, congenital radial head dislocation and Madelung deformity) (Winfeld and Otero, 2016). Authors differentiated between traumatic and congenital deformity by inquiring about the manifestation and evolution of the forearm complaints, presence of trauma in previous medical history and studying clinical and radiographic appearance of both forearms. Patients’ demographics were collected at baseline: age at trauma, age at osteotomy, sex, side of malunion, dominant arm, occurrence of a re-fracture in previous medical history or previous (operative) treatment of forearm malunion.

### Preoperative planning

**Figure 1.**
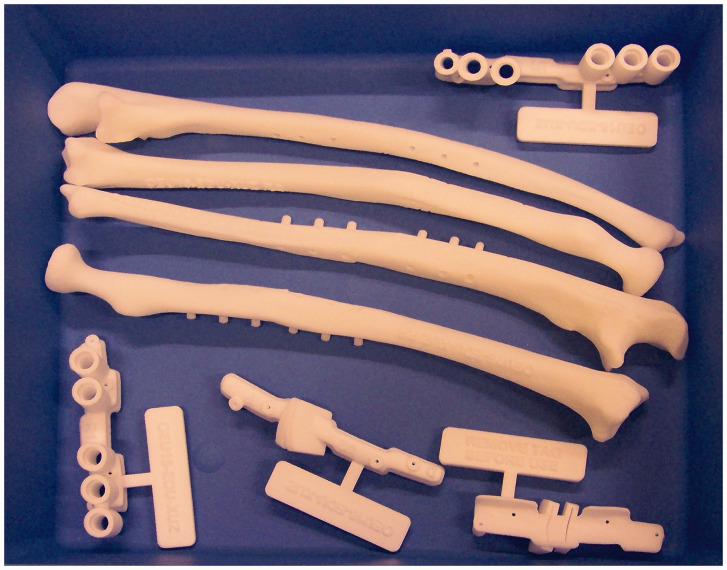
3-D printed patient-specific drilling and cutting guides.

**Figure 2.**
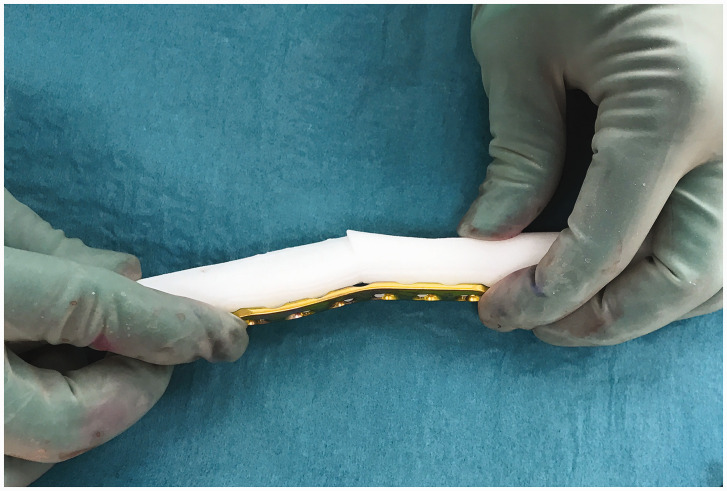
3-D printed real-sized model of the planned correction, used for bending of the plates.

In collaboration with Materialise (Materialise N.V., Leuven, Belgium) planning of the corrective osteotomy was performed according to the following steps: first, a CT scan of both forearms was obtained (0.7 mm slice thickness). Scans were performed with the patient prone with the shoulders in maximal abduction, elbows in maximal extension and forearms as close as possible to neutral (Superman position). A virtual model of the malunited forearm bones was superimposed on a mirrored version of the contralateral forearm bones. Next, the location and degree of deformity were determined. Virtual cutting planes to perform the osteotomy were selected to best match the contralateral side while taking surgical approaches into account. Lastly, patient-specific drilling and cutting guides were 3-D-printed and sterilized to be used during surgery (Figure 1). The drilling guides were designed with the rotational and angular correction built in so that once the osteotomies are completed, the placement of screws determines the correction (Storelli et al., 2015). Also, real-sized models of the forearm bones of the preoperative situation and planned correction were 3-D-printed and used for orientation during surgery and, if needed, for bending of the plates (Figure 2).

### Surgical approach

**Figure 3.**
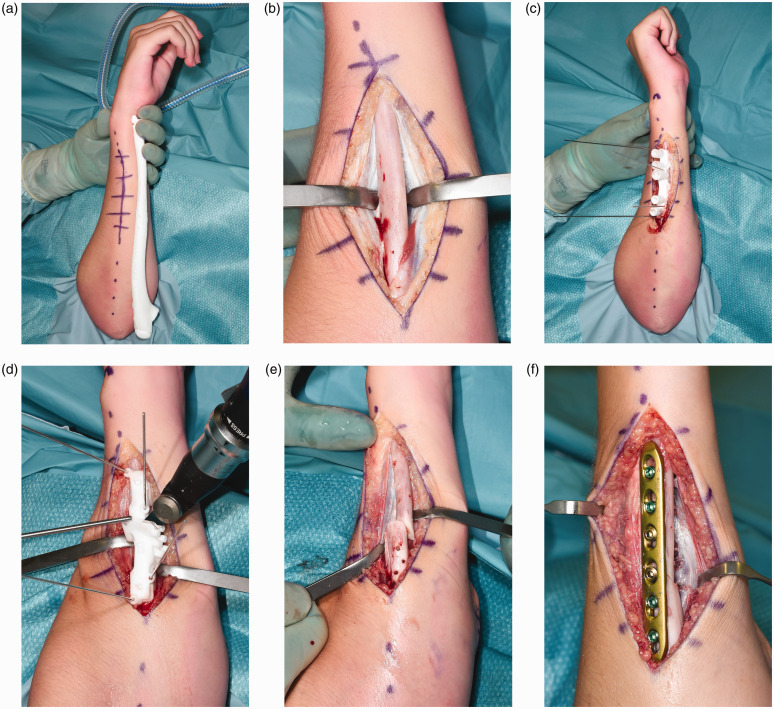
(a) Real-sized model of the preoperative ulna (for orientation). (b) Surgical approach to the ulna. (c) Positioning of the patient-specific drilling guide (for screw positioning). (d) Positioning of patient-specific cutting guide (for corrective osteotomy cut). (e) Corrective osteotomy of the ulna. (f) Plate osteosynthesis of the ulna.

The radius was exposed using a volar Henry approach. Precise positioning of the patient-specific guides was realized by obtaining a wide exposure, searching for recognizable bony landmarks, comparing the intra-operative guide fitting with the 3-D-printed bone models as reference, and use of fluoroscopy. The drilling guide was fixed to the radial shaft with 1.25 mm K-wires to direct correct positioning of the screw holes. The osteotomy cutting guide was then positioned using the same K-wires, and the bone cut was made using an oscillating saw. Next, the ulna was approached in the interval between the flexor and extensor carpi ulnaris, and the planned ulnar osteotomy was performed in a similar manner (Figure 3). Subsequently, the planned correction was performed by positioning the bone segments in a manner such that the previously drilled screw holes aligned. Internal fixation was accomplished using a 3.5-mm, 6-holes locking compression plate (DePuy Synthes Products, Inc., Warsaw, IN, USA). Lastly, the correction and the plate osteosynthesis of the radius were performed in an identical manner. No patient-specific plates or bone grafts were used. After completing the osteosynthesis, range of motion and distal radioulnar joint (DRUJ) stability were tested.

If there was unsatisfactory pro-supination after corrective osteotomy, radioulnar osseous impingement was excluded, and a further release of the interosseous membrane (IOM) was performed if necessary. The IOM was routinely partially released at the level of the osteotomies. The extent of the partial release was what was required to allow the patient-specific drilling and cutting guides to fit around the radius and ulna. If there was persistent impairment in pronation or supination, respectively, the dorsal or volar DRUJ capsule could be released. Kleinman et al. previously stated that a predictable loss of forearm supination will result from post-traumatic contracture of the oblique folds of the redundant volar capsule, and pronation loss can result from similar pathology of the dorsal capsule (Kleinman and Graham, 1998).

Volar DRUJ release was performed by a ‘silhouette’ resection of the volar DRUJ capsule to eliminate pathological thickened tissue that prevents normal forearm rotation (Garcia-Elias and Hagert, 2010).

Through a volar approach and after identifying the position of the triangular fibrocartilage complex by fluoroscopy, the DRUJ was approached through an interval between the ulnar neurovascular bundle and the flexor carpi ulnaris tendon. The neurovascular bundle was retracted radially, the volar radioulnar ligament was identified and protected with great care, and then a volar ‘silhouette’ resection of the DRUJ capsule was performed to completely excise the thickened elements of the capsule itself while protecting the articular surfaces of the distal ulna and distal radius sigmoid notch from injury, as described by Kleinman and Graham (1998).

### Postoperative management

**Figure 4.**
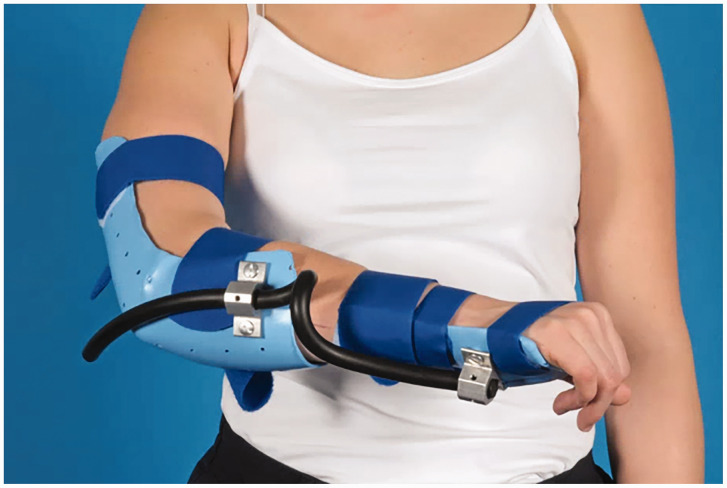
Dynamic bracing in pro- or supination (depending on deficit).

Postoperative management was patient-specific. If full pro-supination was achieved intraoperatively, patients received a compression bandage. If there was a supination deficit, patients received a cast in maximum supination for 2 weeks and vice versa for pronation deficits. Afterwards, treatment was functional (with restrictions for lifting until radiographic consolidation). Patients underwent physiotherapy and were referred to a physical medicine and rehabilitation physician. If full pro-supination was not achieved, dynamic bracing in pro- or supination (depending on the deficit) was used (Figure 4).

### Outcome measures

Our primary outcome was the gain in pro-supination at 12 months follow-up measured with a universal goniometer, using the method as prescribed by the American Society of Hand Therapists (MacDermid et al., 2015). We used a 180° clear plastic, flexible protractor goniometer with two movable 20 cm arms. One arm of the goniometer was lined up parallel to the upper arm of the patient, the other arm was placed parallel to the distal third of the forearm.

Our secondary outcomes were patient-reported outcome measures: the QuickDASH questionnaire (11 items, range 0–100), patient-reported numerical rating scale (NRS) scores for pain and appearance (range 0–10: higher score indicates more pain or poorer cosmetic appearance), maximal grip strength (best of three efforts) using a JAMAR-dynamometer (J.A. Preston Corporation, New York, NY, USA); and the occurrence of complications. Functional outcomes were measured by two non-blinded authors independently (EE and JC). Two separate measurements (of pro-supination) were performed at each follow-up, and averages of both measurements were used. At final follow-up at 12 months after surgery, NRS score for treatment satisfaction was reported (range 1–5: higher score indicates more satisfaction). Outcomes were collected at baseline, 6 months and 12 months follow-up.

### Radiographic analysis

One author (KR) measured angular deformity of the radius and ulna in reference to the contralateral forearm using radiographs in the same forearm position. By superposition of the outlines of the affected and contralateral forearm bones, we determined the location of maximal deformity and angular deformity in both planes (Nagy et al., 2008). The maximum deformity angle was calculated from two measurements of angular deformity – one on the anteroposterior and one on the lateral radiograph, which represented the true angular deformity (Oka et al., 2019). Hereby, deformity was reported as one calculated finding – the true angulation, which increases the comparability of fracture characteristics (Nagy et al., 2008; Roth et al., 2014). Radiographic angulation was re-measured in all cases by a different author (EE) to assess reproducibility.

### Statistics

Outcomes were tested for normality by using the Shapiro–Wilk normality test. We reported medians and interquartile range (IQR) for non-parametric variables and means and 95% confidence intervals (CI) for normally distributed variables. *P*-values < 0.05 were considered statistically significant. Intra-class correlations were calculated to compare reliability of pro-supination and radiographic angulation measurements between observers. Differences between the preoperative and the postoperative ranges of motion, patient-reported outcomes, grip strength and NRS pain and appearance scores were determined using the related samples Wilcoxon signed-rank test due to the small sample size of 15 patients.

Analysis of variance (ANOVA) was performed to assess the relationship between the gain in pro-supination after corrective osteotomy and clinically relevant factors. Subgroups were created for: age at injury ( < 10 years versus ≥ 10 years), time from trauma until corrective osteotomy ( < 1 year versus ≥ 1 year), severity of angular deformity ( < 20° versus ≥ 20°), and severe versus moderate preoperatively impaired pro-supination ( < 69° versus ≥ 69°). Severely impaired pro-supination was defined as an arc of less than 69° of pro-supination, which was based on the necessary arc of 103° (SD 34°) (Sardelli et al., 2011) and subtracting one standard deviation. This equalled 69° (Sardelli et al., 2011). Early corrective osteotomy was defined as being performed within 1 year after trauma. ANOVA was used as exploratory analysis to determine factors associated with a greater gain in pro-supination after 3-D corrective osteotomy. A p-value of < 0.10 was used as threshold during ANOVA to determine which factors progressed to the more-definitive multi-variate linear regression analysis. Next, multivariate linear regression analysis was performed to assess the effect of each factor on a continuous scale while correcting for baseline pro-supination, as we assumed that baseline pro-supination would definitely influence outcome.

## Results

Fifteen participants with a malunited paediatric diaphyseal both-bone forearm fracture with symptomatic impairment in pro-supination were included between October 2016 and July 2018. All patients underwent 3-D-planned corrective osteotomies of both radius and ulna. All surgeries were performed by two surgeons operating together (JC and FS). Patient demographics are presented in the Supplementary Table S1.

Mean age of these patients at trauma was 9.6 years (range 4.0–17.6) and a mean interval between trauma and corrective osteotomy of 5.9 years (range 0.4–12.4). The mean age at osteotomy was 15.5 years (range 10.2–23.3). There was a mean preoperative true radial angulation of 20° (range 11°–31°) and true ulnar angulation of 15° (range 6°–27°).

The interrater reproducibility of the radiological assessment showed an intra-class correlation of 0.78 (CI 0.34–0.93) for the radius and 0.90 (CI 0.71–0.97) for the ulna. Intra-operatively, additional soft tissue releases were performed in four out of 15 patients: there was persistent impairment in supination in Patient 3, 4 and 15, who underwent an additional release of the volar DRUJ capsule. There was persistent impairment in pronation in Patient 8, who underwent an further release of the IOM. There were no additional releases of the dorsal DRUJ capsule. We did not encounter instability of the DRUJ after corrective osteotomy that required additional procedures.

Directly after surgery, a cast in maximum pro- or supination was required in 10 patients. Patient 2 and 8 received a cast in maximum pronation; Patient 3, 4, 5, 9, 12, 13, 14 and 15 received a cast in maximum supination. After 2 weeks the casts were removed, and patients received a dynamic removable splint in maximum pro- or supination. From 2 to 6 weeks postoperatively, the dynamic splint was worn as much as possible and only removed for daily exercises with the physiotherapist. After 6 weeks the dynamic splint was used as a night splint for 3–6 months based on the function. In patients who did not receive a cast in maximum pro- or supination postoperatively (patients 1, 6, 7, 10 and 11), full pro-supination was not maintained, and they too were treated by dynamic splinting. Patient 1 and 6 received a dynamic splint in maximum pronation, while Patient 7, 10 and 11 received a dynamic splint that was alternatively used in maximum pro- and supination.

### Primary outcome

Preoperatively, there was a mean pro-supination of 67° (CI 55°–78°) on the affected side and 153° (CI 148°–158°) on the opposite side. Thus, the affected side had a mean pro-supination of 44% (CI 36%–51%) of the contralateral side preoperatively. At 6 months follow-up there was a mean pro-supination of 118° (CI 105°–130°) resulting in a mean gain in pro-supination of 51° (CI 38°–64°), achieving 78% (CI 71%–85%) of the contralateral side. At 12 months follow-up there was a mean pro-supination of 128° (CI 118°–139°), resulting in a mean total gain of 62° (CI 50°–74°). Contralaterally, there was a mean pro-supination of 150° (CI 144°–155°) at 12 months follow-up. Thus, patients achieved 85% (CI 80%–91%) of contralateral range of motion. The data of individual patients are in supplementary Table S2.

### Predictors for greater functional gain

Outcomes of exploratory analysis (ANOVA) for factors associated with greater gain in pro-supination after 3-D corrective osteotomy are presented in Table 1. Multivariate linear regression analysis revealed that predictors of superior gain in pro-supination at 12 months follow-up after 3-D corrective osteotomy were severe preoperative impairment in pro-supination (*p* = 0.006), shorter time until corrective osteotomy (*p* = 0.03) and substantial angular deformity of the radius (*p* = 0.04).

**Table 1.** Association between clinical/radiological factors and postoperative gain in pro-supination.

Factors	Number of patients	Gain in pro- supination (°)^a^	*p*-value
Age at trauma			
<10 years	10	59 (48–70)	0.53
10 years or more	5	48 (26–108)	
Age at osteotomy			
<13 years	6	71 (50–92)	0.17
13 years or more	9	55 (38–72)	
Time until osteotomy			
<1 year	3	83 (13–152)	0.06
More than 1 year	12	56 (45–68)	
Angulation of radius			
<20°	8	50 (36–64)	0.02
20° or more	7	75 (57–93)	
Angulation of ulna			
<20°	13	63 (49–77)	0.57
20° or more	2	53 (15–91)	
Pre-op pro-supination			
<69°	8	70 (50–89)	0.14
69° or more	7	53 (37–68)	

a Gain in pro-supination data presented as mean (95% confidence interval).

### Secondary outcomes

3-D corrective osteotomy provided a statistically significant and clinically relevant improvement of the QuickDASH score from 32 (15–38) at baseline to 2 (0–11) at final follow-up (*p* = 0.01) (Franchignoni et al., 2014). Differences in grip strength were not statistically significant (*p* = 0.90). Excellent scores for patient satisfaction were reported: 10 out of 15 patients were very satisfied, four were satisfied; one was neutral and none were unsatisfied or very unsatisfied. Secondary outcomes are presented in Table 2.

**Table 2.** Secondary outcomes before surgery and at 6 - and 12-month follow up.

Outcome measures	Preoperative	Post operative (months)	
		6	12
QuickDASH score	32 (15–38)	14 (11–15)	2 (0–11)
Grip strength (%)*	93 (84–103)	82 (66–98)	93 (88–98)
NRS pain score	3 (0.5–6.5)	0 (0–6)	0 (0–3)
NRS cosmetic score	5 (2–6.5)	5 (2–7)	5 (4–5)
NRS satisfaction score	**—**	**—**	5 (4–5)

Data presented as score (interquartile range) in all except for grip strength; grip strength data presented as percentage of the contralateral side (range).

QuickDASH: Disabilities of Arm, Shoulder and Hand score.

Numerical rating scale (NRS) score (range 1–5: higher score indicates better cosmetics or more satisfaction).

### Complications

Ulnar plate removal was performed in one case. One patient had a delayed union. There was a transient neuropraxia of the superficial radial nerve in one patient.

## Discussion

Our patients had a mean improvement in pro-supination from 67° (44% of contralateral) preoperatively, to 128° (85% of contralateral) at 12 months follow-up. Hereby, a greater gain in pro-supination was seen if there was a shorter interval between trauma and corrective osteotomy, substantial angular deformity of the radius and severely impaired pro-supination. Furthermore, in our series 3-D corrective osteotomy provided high patient satisfaction, a decrease in pain score and a clinically relevant improvement in the QuickDASH without the occurrence of any serious complication.

Previously, 3-D corrective osteotomies for paediatric malunited both-bone forearm fractures due to symptomatic impairment in pro-supination have been described in seven studies with a total of 34 patients (Bauer et al., 2017; Byrne et al., 2017; Kataoka et al., 2013; Miyake et al., 2012; Murase et al., 2008; Oka et al., 2019; Walenkamp et al., 2015). Four of the seven studies were included in an individual participant data meta-analysis (Roth et al., 2017) and reported a mean gain in pro-supination of 84° (from 62° to 146°) in 16 patients who underwent 3-D corrective osteotomy. Afterwards, Byrne et al. (2017) reported a mean gain in pro-supination of 61°, from 115° to 176°, in five patients. Bauer et al. (2017) performed 3-D corrective osteotomies for post-traumatic paediatric forearm deformities with impaired pro-supination in 10 patients, leading to a gain in pro-supination of 53°, from 85° to 138°. Oka et al. (2019) reported a mean gain in pro-supination of 47°, from 115° to 162°, in three patients (patients 9–11) after 3-D corrective osteotomy.

Roth et al. (2017) found a greater gain in pro-supination if corrective osteotomy was performed within 1 year after trauma for a mean gain of 93° versus 61°. In our patients, three out of 15 participants underwent corrective osteotomy within 1 year after trauma, and a greater gain in pro-supination was realized (83° versus 56°). Based on our experience, few patients undergo corrective osteotomy within 1 year after trauma, because preferred treatment starts with conservative management and awaits the effect of remodelling and/or physiotherapy. Therefore, an interval until osteotomy of up to 2 years may be considered as an early corrective osteotomy.

In our patients, severe preoperative limitation in pro-supination was a predictor for greater functional gain. This is in line with previous reported series (Roth et al., 2017). Our current study showed that severe angular deformity of the radius was associated with greater gain in pro-supination after 3-D corrective osteotomy. A clear relationship between forearm shaft malunion and significant impairment in pro-supination has been established (Bowman et al., 2011). Previously, in two cadaveric studies, it was demonstrated that angular deformities of 10° resulted in minimum limitation of pro-supination, whereas 20° of angulation caused an important loss of pro-supination, especially in middle-third deformities (Matthews et al., 1982; Tarr et al., 1984).

The value of the conservative treatment of a paediatric forearm malunion is unclear. Until the effect and role of conservative treatment is clear, we recommend considering corrective osteotomy if there is unsatisfactory improvement after conservative treatment. The additional costs for the 3-D planning, patient-specific cutting and drilling guides, and 3-D printed real-sized bones of the radius and ulna is approximately 4.000 Euro per case.

This study has several limitations. The number of patients is relatively small, and there were no control patients with a conventional corrective osteotomy using two-dimensional radiographic planning without patient-specific 3-D printed surgical guides. Also, investigators were not blinded for the side of the surgery during functional evaluation (due to visible scar). Another limitation of the study is that rotational deformity was not assessed.

## Supplemental Material

sj-pdf-1-jhs-10.1177_17531934211029511 - Supplemental material for Outcomes of 3-D corrective osteotomies for paediatric malunited both-bone forearm fractures Click here for additional data file. Supplemental material, sj-pdf-1-jhs-10.1177_17531934211029511 for Outcomes of 3-D corrective osteotomies for paediatric malunited both-bone forearm fractures by Kasper C. Roth, Eline M. van Es, Gerald A. Kraan, Jan A. N. Verhaar, Filip Stockmans and Joost W. Colaris in Journal of Hand Surgery (European Volume)
