# Supplementary material for: Outcomes of 3-D corrective osteotomies for paediatric malunited both-bone forearm fractures
Source: J Hand Surg Eur Vol. 2021 Jul 14;47(2):164–71. doi: 10.1177/17531934211029511 (PMC8801669; doi:10.1177/17531934211029511)
Supplement: sj-pdf-1-jhs-10.1177_17531934211029511 - Supplemental material for Outcomes of 3-D corrective osteotomies for paediatric malunited both-bone forearm fractures [file sj-pdf-1-jhs-10.1177_17531934211029511.pdf]

Table S1: Individual participant data (Demographics)

Patient number	Age at trauma	Time until osteotomy	Sex	Side	Hand Dominance	Re-fracture	Previous treatment	Angulation of Radius	Angulation of Ulna
1	7.1	12.8	F	R	R	Y	C	17.0	19.8
2	9.7	9.8	F	R	R	Y	C	23.9	12.0
3	7.6	6.0	M	L	R	Y	O	14.3	6.0
4	5.0	9.6	F	L	R	Y	C	10.6	26.4
5	8.2	3.4	M	L	L	N	C	22.8	15.6
6	9.7	0.9	F	L	R	N	C	26.2	27.1
7	17.6	0.4	M	L	R	N	C	20.4	19.4
8	14.0	8.6	M	L	L	Y	O	18.2	13.4
9	10.7	9.3	F	L	L	N	C	31.1	12.0
10	13.7	0.7	M	L	R	Y	C	30.9	18.6
11	4.0	6.2	M	R	R	Y	C	22.2	11.4
12	7.4	4.7	M	L	L	Y	O	15.6	6.3
13	12.1	11.2	F	R	R	Y	CO	13.5	9.5
14	9.0	3.2	F	R	L	N	O	13.4	7.2
15	14.8	1.8	M	R	R	N	O	14.9	17.7
Average	9.6	5.9	-	-	-	-	-	19.7	14.8

R/L: Right/Left; F/M: Female/Male; Y/N: Yes/No, C/O: Conservative/Operative; CO: Corrective Osteotomy.

Age at trauma and time until osteotomy data presented as year. Angulation of radius and angulation of ulna in degrees

Table S2: Individual participant data (Functional outcomes)

Patient number	Pro-supination									
	Pre-operative			Postoperative follow up (months)						
				6			12			
	Affected side	Contralateral side	Percentage of contralateral side	Affected side	Percentage of contralateral side	Gain	Affected side	Contralateral side	Percentage of contralateral side	Gain
1	43/18	65/78	42	25/65	67	30	30/70	58/75	75	40
2	30/18	63/90	31	20/80	67	53	38/75	58/83	80	65
3	64/3	70/74	47	48/48	63	28	55/53	65/78	75	41
4	53/-4	66/80	34	33/45	51	29	50/55	68/80	71	56
5	62/10	74/83	46	60/65	79	54	65/75	70/85	90	68
6	23/63	63/90	56	48/80	80	43	53/83	58/85	95	50
7	5/35	75/85	25	63/80	89	103	55/85	65/85	93	100
8	25/53	75/78	51	38/73	72	33	38/80	75/75	78	40
9	45/43	63/90	57	58/90	97	60	58/88	60/88	98	58
10	43/25	72/100	39	60/90	97	83	68/98	73/103	94	98
11	43/5	80/88	28	39/60	76	69	73/98	78/83	103	88
12	55/15	60/83	49	39/80	76	48	70/78	63/85	90	78
13	43/65	78/83	67	50/63	91	33	65/85	80/83	91	28

14	65/20	65/83	58	46/68	79	38	78/70	68/83	92	48	Pro-
15	50/-15	60/85	24	47/53	84	95	68/75	58/85	91	70	
Average	43/24	68/85	44	45/69	78	51	57/78	66/84	85	62	

supination: pronation/supination

Pronation/supination and gain data presented as degrees
